# Whole-Genome Sequence of a Unique *Elioraea* Species Strain Isolated from a Yellowstone National Park Hot Spring

**DOI:** 10.1128/MRA.00907-19

**Published:** 2019-10-31

**Authors:** Sydney Robertson, Robert F. Ramaley, Terry Meyer, John A. Kyndt

**Affiliations:** a College of Science and Technology, Bellevue University, Bellevue, Nebraska, USA; b Department of Biochemistry and Molecular Biology, UNMC, Omaha, Nebraska, USA; c Department of Chemistry and Biochemistry, The University of Arizona, Tucson, Arizona, USA; University of Delaware

## Abstract

The genus *Elioraea* has only one species characterized microbiologically and two genomes sequenced. We have sequenced the genome of a unique *Elioraea* strain isolated from Yellowstone National Park and found it to be a distinct new species. *Elioraea* is suggested to be a member of the aerobic anoxygenic photosynthetic bacteria.

## ANNOUNCEMENT

*Elioraea* is an alphaproteobacterium belonging to the *Acetobacteraceae* family. The first named species of the genus, Elioraea tepidiphila, was isolated from a hot spring in the Azores (1). Elioraea tepidiphila is facultatively chemolithoorganotrophic and grows strictly aerobically, with a slightly thermophilic optimal temperature. Elioraea tepidiphila DSM17972 was designated the type strain, and since then, only 2 other species of the *Elioraea* genus have been identified. Strain YIM 72297 was isolated from a geothermal hot spring in China, and strain PF-30 was isolated from rice paddy soil in South Korea. With only one species description published and only two genome sequences deposited in GenBank (those for DSM17972 and YIM72297), very little is known about the interspecies relationships within this genus.

The new isolate was obtained from the runoff channel of Octopus Hot Spring in Yellowstone National Park, with a sampling point temperature of 50°C. The isolate was purified by streaking on solid *Thermus* medium with 0.3% tryptone and 0.3% yeast extract (2). A partial 16S rRNA gene was sequenced and showed high similarity to Elioraea tepidiphila (99%, 436/438 bp). We designated this new isolate *Elioraea* sp. strain Yellowstone and sequenced its genome to further characterize the species.

We isolated DNA from a living culture grown on *Thermus* medium with 0.3% yeast extract and 0.2% fructose and ribose. Genomic DNA was isolated using the GeneJET DNA purification kit (Thermo Fisher Scientific). DNA analysis using a Qubit fluorometer and a NanoDrop spectrophotometer showed a 260/280 ratio of 1.79. The sequencing library was prepared using the Illumina Nextera DNA Flex library prep kit. The genome was sequenced with an Illumina MiniSeq platform using 500 μl of a 1.8 pM library. Paired-end (2 × 150 bp) sequencing generated 5,851,796 reads and 478.2 Mbp. Quality control of the reads was performed using FastQC within BaseSpace version 1.0.0 (Illumina), using a k-mer size of 5 and contamination filtering. We assembled the genome *de novo* using SPAdes version 3.10.0 (3) through PATRIC (4). This assembly yielded 358 contigs (>300 bp), the largest being 102,200 bp long, with an *N*_50_ value of 23,078 bp. The genome was 3,824,571 bp long (125× sequencing coverage), and the GC content was 72.4%. Using EvalG within PATRIC (4), which uses a reimplementation of the CheckM algorithm (5), we found that the genome had a completeness of 100%. The genome was annotated using the RAST tool kit (RAST*tk*) (6) within PATRIC (4). This showed our strain to have 3,958 coding sequences, and 48 RNAs were identified.

The genomes of the *Elioraea* species were analyzed for biosynthetic pathways of photosynthetic pigments, and it was found that all three species had genes for the photosynthetic reaction center, PufBALMC, in spite of the fact that both *E. tepidiphila* and *E*. *tepidiphila* Yellowstone were reported to be nonpigmented. Because previously characterized *Elioraea* sp. strains are known to only grow aerobically (1), it is most likely a member of the aerobic anoxygenic photosynthetic bacteria (AAP) (7). Other AAP genera related to *Elioraea* are *Rubritepida*, *Paracraurococcus*, *Acidisphaera*, and *Acidiphilium*.

**FIG 1:**
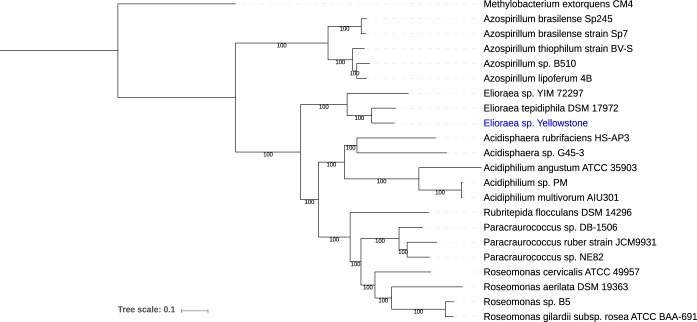
Phylogenetic tree of *Elioraea* whole-genome comparison to its closest relatives. The phylogenetic tree was generated using the codon tree method within PATRIC (4), which used PGFams as homology groups and analyzed 233 aligned proteins and coding DNA from single-copy genes using RAxML (9, 10). iTOL was used for the tree visualization.

A JSpecies comparison (8) of average nucleotide identity (ANI) between this *Elioraea* sp. genome and the two other *Elioraea* sp. genomes gave 85.0% identity with Elioraea tepidiphila DSMZ 17972 and 76.8% with *Elioraea* species strain YIM72297. The ANI values for *Elioraea* sp. strain Yellowstone are clearly below the proposed 95% cutoff for genome definition of a species (8), suggesting it belongs to a unique species. The ANI also showed that *Elioraea* species are not very closely related to the other AAP mentioned above (<72% identity). Phylogenetic analysis of the *Elioraea* sp. strain Yellowstone genome using RAxML within PATRIC (9, 10) showed *E. tepidiphila* DSM17972 to be the closest relative, followed by *Elioraea* sp. strain YIM72297 as a distinct genus within the *Acetobacteraceae* (Fig. 1).

### Data availability.

This whole-genome shotgun project has been deposited at DDBJ/ENA/GenBank under the accession number VIKA00000000. The version described in this paper is version VIKA01000000. The raw sequencing reads have been submitted to the SRA, and the corresponding accession number is SRR9613384.
